# Health Care Costs and Participation in a Community-Based Health Promotion Program for Older Adults

**Published:** 2010-02-15

**Authors:** Charles Mayer, Barbara Williams, Edward H. Wagner, James P. LoGerfo, Allen Cheadle, Elizabeth A. Phelan

**Affiliations:** University of Washington Health Promotion Research Center, Department of Health Services, School of Public Health and Community Medicine, University of Washington Department of Family Medicine, Seattle; University of Washington, Seattle, Washington; Group Health Cooperative of Puget Sound, Seattle, Washington; University of Washington Departments of Medicine and Global Health, Seattle, Washington; University of Washington Health Promotion Research Center, Seattle, Washington; University of Washington Health Promotion Research Center and University of Washington, Seattle, Washington

## Abstract

**Introduction:**

EnhanceWellness (EW) is a community-based health promotion program that helps prevent disabilities and improves health and functioning in older adults. A previous randomized controlled trial demonstrated a decrease in inpatient use for EW participants but did not evaluate health care costs. We assessed the effect of EW participation on health care costs.

**Methods:**

We performed a retrospective cohort study in King County, Washington. Enrollees in Group Health Cooperative (GHC), a mixed-model health maintenance organization, who were aged 65 years or older and who participated in EW from 1998 through 2005 were matched 1:3 by age and sex to GHC enrollees who did not participate in EW. We matched 218 EW participants by age and sex to 654 nonparticipants. Participants were evaluated for 1 year after the date they began the program. The primary outcome was total health care costs; secondary outcomes were inpatient costs, primary care costs, percentage of hospitalizations, and number of hospital days. We compared postintervention outcomes between EW participants and nonparticipants by using linear regression. Results were adjusted for prior year costs (or health care use), comorbidity, and preventive health care-seeking behaviors.

**Results:**

Mean age of participants and nonparticipants was 79 years, and 72% of participants and nonparticipants were female. Adjusted total costs in the year following the index date were $582 lower among EW participants than nonparticipants, but this difference was not significant.

**Conclusion:**

Although EW participation demonstrated health benefits, participation does not appear to result in significant health care cost savings among people receiving health care through a health maintenance organization.

## Introduction

Several health promotion and disease prevention programs designed for older adults have been developed and evaluated for their health benefits and resource use (1-5). These programs focus on improving older adults' general health and encouraging self-management of chronic medical conditions. Specific aspects of health improvement, such as improving mental well-being or increasing physical activity, are often the focus of health improvement and are pursued because of a client's interest and motivation. These programs connect clients with information and resources that help them address their personal health concerns, build confidence in health care decision making, and increase physical activity. Such health promotion programs for older adults improve health outcomes, and they have demonstrated decreased use of health care resources resulting from participation, which results in decreased health care costs (2,3). However, to our knowledge these studies used self-reported data rather than actual health care costs.

EnhanceWellness (EW, formerly known as Health Enhancement Program, or HEP) targets older people at risk for functional decline. Nurses and social workers meet with community-living elders to help increase physical activity, promote social activity, improve mental health, and enhance self-management of chronic conditions to improve health and functioning. In a 1998 randomized controlled trial, EW participants increased their physical activity, decreased their use of psychoactive medications, and decreased their number of hospital days (2). After that study, senior centers in the Seattle, Washington, area began implementing EW, and enrollees of Group Health Cooperative (GHC), a consumer-governed, nonprofit health care system that provides both health care and medical coverage, started participating in the program. A follow-up study conducted in 2002 evaluated the program as it operated in the community, outside the controlled setting of a randomized trial (5). This study also demonstrated significant benefits, including a reduction in disability risk factors, improvement in health status, no decrease in functional status, and no increase in self-reported health care use.

EW has been confused with the EnhanceFitness Program (EF) because of their similar names and the fact that both have been studied in a similar older adult population (6,7) However, the programs are distinct: EF is a group exercise program, whereas EW is a comprehensive, participant-centered wellness program that includes a health assessment, a tailored health plan, and motivational support to achieve a self-chosen goal. EW participants, if desired, may include regular physical activity and join EF, a covered benefit for GHC members. Less than 10% of GHC members typically participate in both programs, although not necessarily simultaneously (M. Thompson, oral communication, December 2008).

Although health benefits and a reduction in hospital days have been demonstrated, EW's effect on health care use and costs has not been previously analyzed. The availability of comprehensive cost and use data for GHC members made studying these questions with GHC members who had participated in EW attractive. We hypothesized that participation in EW would lower overall health care costs, via reductions in costly forms of health care use (especially hospitalizations).

## Methods

### Study setting

EW is offered at community centers, many of which are senior centers, located in the greater Puget Sound region. Senior Services, a private nonprofit organization with 250 employees established in 1967, operates EW. Nearly all nurses and social workers in EW programs in King County are employed either by Senior Services or by the hosting EW sites. The main sources of funding for Senior Services for EW programs in King County are the Aging and Disability Services of Seattle and King County and the Public Health Department of Seattle and King County. Office space and supplies are often donated by hosting sites. Participants are asked to make a donation at the time of graduation but this amount covers only a small amount of actual EW costs. Senior Services estimated that the cost to administer EW at its King County sites in 2004 was $400 per participant per year. Although EW has been disseminated beyond King County, Washington, we restricted our study to King County, where GHC is based (8).

GHC is a consumer-governed, mixed-model health maintenance organization (HMO) with more than 500,000 members in the Pacific Northwest; according to our research, approximately 65,000 members are aged 65 years or older, and 27,900 reside in King County. Health outcomes and cost data are available and complete for all GHC members, regardless of whether they receive their care at a GHC-owned health care facility. GHC health care use and cost data have been studied and validated (9), and we used these data to capture our outcomes data. The institutional review boards of the University of Washington and GHC approved the study protocol.

### Participants

We chose our sample from GHC members who were aged 65 years or older, resided in King County, and voluntarily participated in EW from March 15, 1998, through April 15, 2005. From this group, we selected participants who were continuously enrolled in GHC for at least 1 year before and 1 year after the first day of their EW enrollment. The date of EW enrollment (ie, the first day an EW participant signed a consent form, formally agreeing to participate in the program) was defined as the "index date." We excluded enrollees who had been in a long-term–care facility during the year before the index date because of the high costs involved that would have skewed the overall results.

Each EW participant was age- and sex-matched to 3 GHC members who had not participated in EW ("nonparticipants"). Nonparticipants were assigned an index date that corresponded to the index date of the EW participant to whom they were matched, creating comparable pre-index and postindex enrollment periods. Inclusion criteria for nonparticipants were identical to criteria for EW participants. Our analysis included comparisons between 218 EW participants and 654 matched nonparticipants.

### Intervention

EW has been described in detail elsewhere (2,3). Briefly, after EW clients complete the program's health intake questionnaire, which assesses risk factors for functional decline, they meet with a social worker or nurse for approximately 1 hour to discuss personal health concerns, review the findings of the questionnaire, and identify personal health goals. Clients develop strategies for improving health and make "health action plans." They are encouraged, but not required, to seek out health and community services when needed. These services may include appointments with primary care providers, medical specialists, social services, or mental health services, or participation in an organized exercise program. Clients often need follow-up appointments with the nurse or social worker, either in person or by telephone. The recommended minimum time for program participation was 1 year until November 2003, at which point the recommended minimum time was reduced to 6 months.

### Outcome measures

Total health care costs during the year following the index date was the primary outcome measure. Total costs included inpatient, primary care, and nonprimary care outpatient costs. Nonprimary care outpatient costs consisted of outpatient specialty care, outpatient mental health, emergency department care, outpatient pharmacy, outpatient laboratory, outpatient radiology, long-term care, and drug and alcohol treatment costs. Secondary outcomes were inpatient and primary care costs, percentage of hospitalizations, and number of hospital days. All cost data were captured from the GHC cost accounting system previously described (6,9).

### Data analysis

Participation (yes/no) in EW was our main predictor of interest. We included age, sex, prior year health care costs or use (as appropriate), comorbidity, and tendency to use preventive services as covariates in our analyses because these factors typically influence health care use and costs. We assessed comorbidity and chronic disease burden by using the GHC diabetes and heart registries and the Charlson comorbidity index (10). We used the methods of the HMO Research Network, which based its index on the method outlined by Deyo et al (11), with the addition of peripheral vascular disorder procedure codes and outpatient encounters as recommended (10,12,13) to determine our Charlson comorbidity index.

We assessed inclination to use preventive health services by using a preventive services score, which takes into account preventive health services and preventive visits (14,15). This score is the sum of the number of times a study participant received colon cancer screening (fecal occult blood test or flexible sigmoidoscopy), a screening mammogram, prostate cancer screening, an influenza vaccine, or a pneumococcal vaccine during the 2 years immediately preceding the index date (score range, 0-8). If the person had none of the 4 services in the past 2 years, the preventive services score was the number of primary care preventive visits the person had in the past 2 years (maximum, 2).

Median household income and median education were available for analysis at the census block level for more 80% of our participants. These socioeconomic status variables were considered but not included in the final model because their inclusion did not alter our results.

### Statistical analysis

The primary analysis focused on differences in health care costs and use between EW participants and matched nonparticipants. We adjusted all costs to 2005 US dollars by accessing the Medical Care component of the Consumer Price Index for the participant's index year (16). We used 2-tailed *t* tests and χ^2^ tests to compare demographic and health-related characteristics and unadjusted health care cost and use measures for participants and their matched comparisons. We used ordinary least squares linear regression to analyze cost differences, adjusting for covariates; this modeling approach yields unbiased estimates of differences in mean costs when the sample size is large (17). Because the distribution of health care costs is often skewed, as many people have no costs and a few have high costs, we repeated our analysis by using log-transformed costs. All analyses were performed using Stata, version 9.0 (StataCorp LP, College Station, Texas).

## Results

Most EW participants (88%) spent 6 months or longer in the program; approximately 50% spent 12 or more months, and 20% were in the program for more than 2 years. EW participants were identical to nonparticipants in terms of average age (79 y) and sex (72% female) (Table 1). We noted several significant differences between the groups, including a larger comorbidity burden among EW participants, as measured by a higher Charlson comorbidity index and a larger proportion enrolled in the GHC diabetes and heart disease registries. The preventive services score was significantly higher for EW participants, suggesting a stronger tendency to receive preventive services.

Total costs, inpatient costs, percentage hospitalized, and number of hospital days were not significantly different between participants and nonparticipants, either at baseline or in the year after the index date (Table 2). The only significant difference was in unadjusted primary care costs, which were higher by $325 in the EW group (*P* < .001) at baseline and $177 higher in the year after the index date (*P* = .04). After adjusting for age, sex, prior year total costs, preventive services score, Charlson comorbidity index, and presence on the GHC diabetes or heart disease registries, total health care costs in the year after the index date were $582 lower for EW participants than for nonparticipants, but this difference was not significant. The results were unchanged when we used log-transformed costs. There were no differences in inpatient use or primary care use between the 2 groups at baseline or the year after the index date.

## Discussion

We found that, compared with nonparticipants, EW participants had nonsignificantly lower total health care costs and no difference in hospitalizations during the year following EW enrollment. This finding may have resulted from the fact that EW participants in our sample had a significantly larger comorbidity burden than did nonparticipants. Comorbidity is a major driver of hospital costs and total annual costs (18,19). Furthermore, the methods we used to adjust for comorbidity, although widely used, may not have allowed us to fully control for comorbidity differences between study groups (10,11,13)

Many health promotion programs, some designed for the older adult population, have been associated with decreased health risks and decreased health care use (1-3,20-23). Health promotion programs evaluated by Lorig et al and Holland et al most closely resemble the EW program (3,4). These studies evaluated health outcomes and health care use, but neither assessed health care costs. Lorig et al found a significant decrease in hospitalizations and hospital days during their 6-month randomized controlled trial. The average age in this study was 10 years younger than in ours, and the 2 study groups had balanced comorbidities. Conversely, Holland et al did not find a difference in health care use between study groups during their year-long randomized controlled trial of the Health Matters Program in Sacramento, California, a program modeled after EW (4,24). Similar to our analysis, the mean age of participants in Holland's study was 73 years.

There are several differences between our analysis and the original randomized controlled trial that was used to evaluate EW (2). The original trial lasted 12 months, and outcomes were evaluated for the 12 months of program enrollment. After the original trial, EW evolved into a 6-month program, so our analyses included EW participants with varying duration of program participation. For most EW participants, 6 months of EW participation confers favorable effects on disability risk factors (eg, depression, physical inactivity) that are comparable to 12 months of participation (25). However, such reductions in disability risk factors do not appear to translate into lower overall health care costs.

A strength of our analysis was that we reported actual health care costs. To our knowledge, no other analysis of a health-promotion, disability-prevention health resource has used actual cost data. Health care use is often used as a proxy for costs, or alternatively, costs are estimated from claims data (1,22). Furthermore, our health care use data were derived from automated data sources, which are more accurate in assessing health care use and costs than are self-reported data (26,27).

Our study has several limitations. Our study had an observational design, which can result in residual confounding and selection bias. Residual confounding in relation to the comorbidity differences we observed between study groups is likely, although we attempted to adjust for them. Research published after our study ended demonstrated that total annual costs increase with increasing comorbidity and that 4 conditions — hypertension, depression, use of warfarin, and skin ulcers/cellulitis — should be added to the Charlson comorbidity index to accurately predict total annual costs (19). We used the preventive services score to address selection bias related to the potential tendency of more prevention-oriented people to participate in EW. This score has been used in prior research with GHC members but may not have fully accounted for this form of selection bias (14). We also considered using propensity scores to adjust for selection bias but lacked enough covariates to independently predict program participation.

Another limitation was a lack of detail on health care use. In particular, we could not distinguish increased use that may have been prompted by participation in EW (eg, more visits related to health problems identified by EW). Also, apart from EF, we had no information about exercise and other health promotion programs that EW participants may have pursued as a result of their participation in EW. Finally, our sample size of just over 200 EW participants may have been too small to detect meaningful cost differences given the large variances associated with health care cost and use data.

EW improves the health of older adults at risk for functional decline (28). However, we did not find that overall health care costs were significantly reduced by EW program participation.

## Figures and Tables

**Table 1:** Participant and Nonparticipant Demographic Characteristics, Group Health Cooperative/EnhanceWellness, March 1998-April 2005

**Characteristic**	Participants (N = 218)	Nonparticipants (N = 654)	*P* Value
Mean age, y (SD)	78.6 (5.8)	78.6 (5.8)	>.99
% Female	72.5	72.5	>.99
Mean preventive services score (SD)^a^	3.0 (1.2)	2.8 (1.4)	.03
Mean Charlson comorbidity index (SD)^b^	1.0 (1.3)	0.7 (1.2)	<.001
% Listed on GHC diabetes registry	20.6	14.1	.02
% Listed on GHC heart disease registry	43.1	29.2	<.001

a Derived from the sum of the number of times a subject received colon cancer screening (fecal occult blood test or flexible sigmoidoscopy), a screening mammogram, prostate cancer screening, an influenza vaccine, or a pneumococcal vaccine during the 2 years immediately preceding the index date (score range 0-8; higher scores indicate receipt of more preventive services).

b See Methods section for a description of this score. The mean Charlson comorbidity index and the percentage of participants listed on the Group Health Cooperative diabetes and heart disease registries were used to measure comorbidity and chronic disease burden.

**Table 2:** Health Care Costs and Use of Participants and Nonparticipants at Baseline and Year Following Index Date,^a^ Group Health Cooperative/EnhanceWellness Program, March 1998-April 2005

Variable	Unadjusted Results				Adjusted Results	

	Participants (N = 218)	Nonparticipants (N = 654)	Difference	*P* Value^b^	Difference	*P* Value^b^
**Cost, $^c^**						
**Total**						
Baseline	7,047	6,207	840	.20	−582	.58
Year 1	8,091	7,977	114	.91		
**Inpatient**						
Baseline	773	1,116	−343	.29	−804	.22
Year 1	1,334	2,162	−828	.21		
**Primary care**						
Baseline	1,213	888	325	<.001	28	.72
Year 1	1,069	892	177	.04		
**Health care use**						
**No. of hospital days**						
Baseline	0.43	0.40	0.03	.78	−0.15	.56
Year 1	0.83	0.89	−0.06	.80		
**% Hospitalized**						
Baseline	10.6	8.6	2.0	.38	−0.02	.95
Year 1	13.3	13.3	0	>.99		

a The "index date" is date of enrollment (ie, the first day a participant signed a consent form, formally agreeing to participate in the program).

b *P* values for unadjusted results derived from *t* tests; *P* values for adjusted results derived from linear regression (adjusted for age, sex, prior year costs, preventive services score, Charlson comorbidity index, and presence on the Group Health Cooperative diabetes or heart registries).

c Results reported in mean 2005 US dollars.
